# At a crossroads: How can Nepal enhance its community health care system to achieve Sustainable Development Goal 3 and universal health coverage?

**DOI:** 10.7189/jogh.10.010309

**Published:** 2020-06

**Authors:** Ryan Schwarz, Aradhana Thapa, Sudha Sharma, SP Kalaunee

**Affiliations:** 1 Nyaya Health Nepal, Kathmandu, Nepal; 2 Former Secretary at Ministry of Health & Population, Nepal

Nepal has made tremendous health gains over recent decades. In 2010, Nepal was recognized by the United Nations for its impressive progress towards Millennium Development Goal 5, with a 3-fold reduction in maternal mortality rate between 1990 and 2015. During the same period the country halved rates of stunting and achieved all indicators of Millennium Development Goal 4 towards reducing child mortality [1]. These gains are matched with a revitalized commitment to universal health coverage (UHC), including the recent passage of the 2017 National Health Insurance Act and 2018 Public Health Act, both of which will contribute to ensuring Nepal’s constitutional commitment of health care as a human right and progress towards the Sustainable Development Goals (SDGs).

As Nepal looks ahead, policymakers must identify new paths to improve primary health care (PHC) performance. Despite progress, analysis demonstrates persistent challenges with health care access, quality, and equity. The maternal mortality rate stands at 239 per 100 000 live births and the under-5 mortality rate at 39 per 1000 live births [2], with deaths mostly due to preventable causes. Nepal’s progress towards the SDGs is commendable, yet Nepal is not currently on-track to meet targets. For example, Nepal must further enhance its efforts as a 9% annual reduction in maternal mortality is necessary to achieve SDG targets.

Globally, community health care systems make up a core foundation of effective, accessible, and equitable PHC. They are an important and cost-effective component in the realization of UHC, with an economic return of up to 10:1 [3]. Recently, the World Health Organization (WHO) released the first global guidelines on design and implementation of community health worker (CHW) programs [4] by synthesizing global evidence and experience, offering important guidance for countries striving to achieve SDG3 and UHC. In Nepal, community health has been critical to improvements in public health so far, and we here consider how Nepal can further improve its community health system to achieve SDG3 and UHC.

For decades, Nepal has been a leader in community health care. The Female Community Health Volunteer (FCHV) program was developed in 1988 and includes a national network of over 50 000 volunteers. The FCHV program has served as the backbone of Nepal’s community health system and has been the foundation for gains made in recent decades. Today, FCHVs continue to serve as critical pillars for behavior change communication including maternal and child health, and for selected services including family planning, oral rehydration salts, vitamin A distribution, and outreach clinics.

**Figure:**
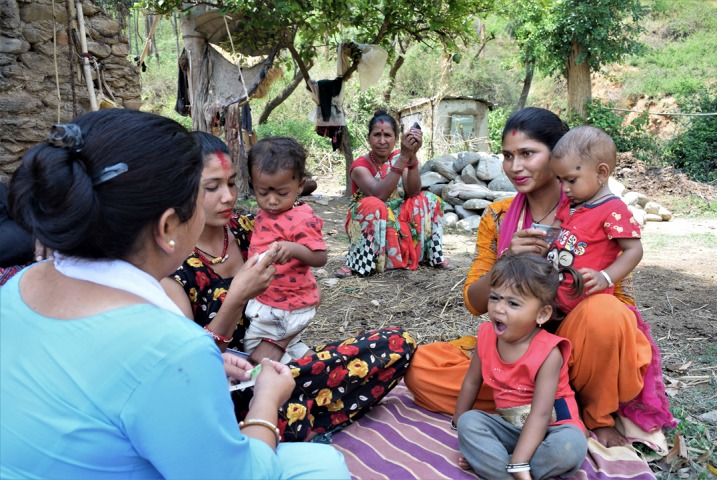
Photo: Community health worker counseling family members in rural Nepal (from Nyaya Health Nepal, used with permission).

However, the FCHV program is now over 30 years old, and there are increasing questions about whether its design and capacity can continue to advance Nepal on its trajectory towards SDG targets [5,6]. For example, high levels of disparity persist in maternal health care with antenatal care, institutional birth, and postnatal care all correlated strongly with maternal educational status – for women with a secondary education, the institutional birth rate is 85%, yet only 38% for women with no education. Similarly, for women in the highest wealth quintile institutional birth is 89%, but 34% for women in the lowest quintile [2]. Despite the important foundation the FCHV program provides, great inequity and barriers to PHC remain.

The WHO guidelines aim to assist governments and stakeholders to improve CHW program design, implementation, and performance [4]. Broadly, the guidelines recommend CHWs be locally-selected, engaged in their community, trained both pre-service and continuously, formally certified, supervised, paid, provided opportunities for career growth, engaged in data reporting, and closely integrated with local PHC facilities. In contrast, Nepal’s FCHV program, among other challenges, is volunteer, has no minimum educational requirement, and has limited supportive supervision, training and career advancement opportunities [7]. WHO guidelines offer Nepal’s policymakers an opportunity to re-evaluate the present system by identifying successful and impactful areas, while also exploring opportunities for improvement to achieve UHC and SDG3 (**Table 1**). As Nepal moves forward, we encourage policymakers to consider these guidelines in planning for the future of its community health system, especially with the introduction of a formal cadre of paid CHWs.

**Table 1:** WHO CHW guidelines and Nepal’s community health care system

	Selected highlights from WHO CHW Guidelines [4]	Performance successes and opportunities for improvement in Nepal’s community health care system [7]
**Selecting, training, and certifying CHWs**		
**Selection**	Minimum education level specified	No minimum education level required
	Require community membership	Yes
	Apply appropriate gender equity to context	Yes
	Do not use age or marital status as criteria	FCHVs must be between 25-45 y of age
**Pre-service training, curriculum, and training modalities**	Pre-existing knowledge accounted for	FCHV training opportunities are challenged by inconsistency, lack of comprehensiveness on content for expected responsibilities, regular updates and continuity, and variation by geography
	Based on CHW role and responsibilities, including training on preventive, promotive, diagnostic, and curative services	
	Balance between theory and practice, with training conducted in the community	
**Certification**	Competency-based formal certification	Yes
**Managing and supervising CHWs**		
**Supportive supervision**	Appropriate CHW to supervisor ratio that enables regular and meaningful support	FCHV supervision is infrequent and inconsistent, and occurs predominantly at health facilities
	Supervisors are trained sufficiently to provide CHWs effective support	Limited training on supervision modalities is provided to PHC staff overseeing FCHVs
	Supervisors utilize observation of service delivery, performance data, and community feedback, to provide CHWs feedback on performance	Limited performance feedback is provided to FCHVs, and when provided is not typically supported by data, service observation, or community feedback
**Remuneration**	CHWs are paid commensurate to responsibilities	FCHVs are volunteer
	CHWs should not be paid exclusively or predominantly via performance-based incentives	FCHVs receive intermittent and inconsistent incentives, limited in scope, with significant variation by geography
**Contracting and career ladder**	For paid CHWs, provide written contract specifying responsibilities, remuneration, and workers’ rights	N/A (not paid)
	Career ladder should be offered to create pathway to other qualifications or role progression	FCHVs have no career advancement opportunities
**Integrating CHWs into health systems and gaining community support**		
**Target population**	Consider population geography, epidemiology, and other barriers, and account for expected workload	Yes
**Collection and data utilization**	CHWs should document services, and collect, collate, and use health data, routinely, including through mHealth platforms	FCHVs are trained, however documentation and utilization of data are inconsistent. All data are on paper registers.
	CHWs should be provided with feedback based upon data collected	Feedback to FCHVs is limited and inconsistent
**Types of CHWs**	Service delivery model should include CHWs with general tasks as part of integrated PHC teams	Yes
**Community engagement**	Involve communities in CHW selection, program promotion, planning, priority setting, and evaluation	Yes
**Mobilization of community resources**	CHWs should identify priority health and social needs of community and mobilize resources and action plans accordingly.	Yes
	Strengthen linkages between community and local health facilities	Yes
**Availability of supplies**	Ensure CHWs have regular and quality-assured health-related goods via health supply chain	FCHV supply chain quality varies geographically, and can be inconsistent and unreliable

WHO – World Health Organization, CHW – community health worker, FCHV – female community health volunteer, PHC – primary health care

CHW programs across the globe employ various models to operationalize the WHO recommendations. Brazil’s Community Health Agents for example, are full-time, salaried, community-based workers supervised by local PHC staff, with a ratio of ~ 1:600. The Community Health Agent program has grown since the 1980s, evolving to meet changing priorities and growing to cover over 200 million lower-income people. Alternatively, Ethiopia provides a compelling example of a “dual-cadre system,” in which Health Extension Workers collaborate with Health Development Army Volunteers. Health Extension Workers are certified, salaried, government employees, with a minimum education requirement, 12-month training, and supervised by local PHC facilities, with a ratio of ~ 1:2500. Health Extension Workers work with Health Development Army Volunteers – part-time community mobilizers – who receive non-financial incentives and support PHC with additional promotive activities, at a ratio of ~ 1:25 [8]. These and other global examples are worth significant consideration.

Nepal is at a crossroads, committed to SDG3 and UHC, and in need of improved PHC to achieve them. Robust CHW programs are cost-effective [3], and can and should be a key component on this path – examples globally, combined with recent WHO recommendations, offer important guidance for Nepal’s next steps. Additionally, an ongoing CHW pilot run under Nepal’s Family Welfare Division is closely aligned with WHO guidelines, including CHWs who are salaried, continuously-trained, with robust supervision and linkages to PHC. Preliminary outcomes are positive, and further data are expected in 2020 [9]. We believe that a cadre of paid, well-supervised CHWs, similar to those employed in the pilot program, could offer important value to the present FCHV network in a model similar to that employed in other settings.

Nepal’s recent transition to federalism, including decentralization of health care administration and policy, will lead to greater local accountability, authority, and locally-owned health care service delivery. Robust CHW programs can help ensure municipalities are able to provide equitable and responsive health care to their constituencies, as part of the country’s priority Basic Healthcare Package, and simultaneously address growing national priorities, including civil registration and vital statistics, and the growing non-communicable disease burden, which the current cadre of volunteers won’t be able to address.

In addition to gaps in the FCHV program (**Table 1**), challenges with other prior community-based cadres illustrate the difficulty of scaling new community health systems in Nepal – politically, financially, and operationally [8]. In particular, supervision and continued training have historically been significant challenges throughout Nepal [7,8]. However, given the newly decentralized health system, and increasingly important localized accountability, paired with a strong commitment to improving PHC and achieving SDG targets, we believe there is a renewed opportunity to employ more robust community-based health care delivery. Additionally, given significant advancements in mobile health technologies to support CHWs since the development of the FCHV program, a cadre with a minimum educational requirement who can more readily utilize such technologies could offer further value to the public health system. Thus, by deploying a paid, robustly-supported cadre, Nepal can continue to leverage the important foundation of the FCHV network, while also making further progress towards the SDGs.

In parallel, Nepal’s SDG roadmap, and recent passage and implementation of national health insurance demonstrates continued commitment to increasing domestic health care financing, which can and should be expanded to cover broader community-based services given high return on investment. Further, investments globally for community health care systems are growing dramatically to match increased recognition and excitement for CHWs as key to UHC [10]. Nepal’s historical leadership in community health care and its own commitment to UHC, paired with a growing ecosystem of stakeholders, should aim to harness similar support and financing.

The Nepal Health Sector Strategy concludes in 2020. As policymakers consider priorities for the next five-year strategy, including opportunities to improve access and quality, we encourage them to consider the WHO recommendations, which highlight the importance of paid, well-supervised, continuously-trained CHWs closely-integrated into PHC systems. In parallel, examples from other successful programs globally, and the ongoing pilot in Nepal, offer important insights. As Nepal looks ahead, robust community health care must be a core pillar of quality PHC to match the capacity Nepal has committed to in achieving SDG3 and UHC.
